# Scores of the Cleft Hearing, Appearance and Speech Questionnaire (CHASQ) in Swedish Participants With Cleft lip and/or Cleft Palate and a Control Population

**DOI:** 10.1177/1055665620952296

**Published:** 2020-09-04

**Authors:** Mia Stiernman, Kristina Klintö, Martin Persson, Magnus Becker

**Affiliations:** 1 Department of Plastic and Reconstructive Surgery, 59565 Skåne University Hospital, Malmö, Sweden; 2 Department of Clinical Sciences, Malmö, 5193 Lund University, Sweden; 3 Department of Otorhinolaryngology, 59565 Skåne University Hospital, Malmö, Sweden; 4 Department of Health and Society, Kristianstad University, Sweden

**Keywords:** patient-reported outcomes, cleft lip and/or cleft palate, appearance satisfaction, speech satisfaction, control population

## Abstract

**Objective:**

The primary aim of this study was to investigate whether there was any difference in scores of the Cleft Hearing, Appearance and Speech Questionnaire (CHASQ) between patients with cleft lip and/or cleft palate (CL/P) and a control population. The second aim was to compare CL/P and control population scores in this study with a British norm CL/P population.

**Design:**

Single-site, cross-sectional study with an age-matched control population.

**Setting:**

Participants were recruited from a hospital, a school, and a sports club. They answered the CHASQ in the hospital or at home.

**Participants:**

Sixty-four participants with CL/P (7-19 years of age) and a control population of 56 participants without CL/P (9-20 years of age).

**Main Outcome Measure:**

CHASQ.

**Results:**

There was no statistically significant difference in satisfaction with cleft-related features between the CL/P and the control population. Participants with CL/P were significantly more satisfied with non-cleft-related features than the control population. Cleft Hearing, Appearance and Speech Questionnaire scores were also similar to earlier established British normative data of a CL/P population.

**Conclusion:**

The results indicated that children and young people with CL/P were as satisfied with their appearance, hearing, and speech as children and young people without CL/P. Swedish CHASQ scores were also similar to British scores.

## Introduction

Health-related quality of life (HRQOL) is defined by the International Society for Quality of Life Research (ISOQOL) as “the functional effect of a medical condition and/or its consequent therapy upon a patient. Health-related quality of life is thus subjective and multidimensional, encompassing physical and occupational function, psychological state, social interaction and somatic sensation” (ISOQOL, 2019). High HRQOL is one of the most important goals in the treatment of cleft lip and/or cleft palate (CL/P) and incorporating patient perspective and Patient-Reported Outcomes (PROs) into cleft care is recommended (Semb et al., 2005; Klassen et al., 2012; American Cleft Palate-Craniofacial Association, 2018). The Cleft Hearing, Appearance and Speech Questionnaire (CHASQ) is a patient-reported outcome measure (PROM) which has been used in clinical research in Europe (Stiernman et al., 2019). Cleft Hearing, Appearance and Speech Questionnaire measures patient satisfaction with hearing, speech, and different features of facial appearance. These aspects of HRQOL are important targets of treatment of CL/P and essential in care evaluation. In a study by Stiernman et al. (2019), health care professionals from across Europe regarded CHASQ useful, short, and easy to use in a clinical setting. It has, however, not yet been evaluated in a control population.

### Earlier Comparisons of PRO Between CL/P and Control Populations on Hearing, Appearance, and Speech

Inclusion of control populations in studies on satisfaction and psychosocial health in patients with a cleft is strongly recommended (Aaronson et al., 2002; Ranganathan et al., 2015; Stock and Feragen, 2016; Stock et al., 2018). This is a prerequisite for being able to draw conclusions about the general level of health within a studied population and where to draw the cutoff for further investigation or intervention.

Patients in their 20s and 30s with bilateral cleft lip and palate (BCLP) have reported similar quantitative satisfaction with hearing as a control population (Oosterkamp et al., 2007). However, in the same study, qualitative results revealed that participants with BCLP had significantly more concerns regarding hearing in noisy environments, partial deafness, and difficulties with high and low sounds than the control population. In an additional quantitative study, participants with unilateral cleft lip and palate (UCLP) were significantly less satisfied with their hearing than the control population without UCLP (Van Lierde et al., 2012).

A narrative review by Stock and Feragen (2016) concluded that most studies, which included a control or reference population without CL/P, found that participants with CL/P reported reasonable levels of satisfaction with appearance. A further study (Feragen et al., 2015), which included analysis of perception of physical appearance, showed similar scores for 16-year-olds with or without CL/P. Participants with CL/P were also reported to be more satisfied than a control population regarding non-cleft-related appearance as well as overall appearance (Berger and Dalton, 2009). However, a number of studies also found that participants with CL/P reported lower satisfaction with facial appearance than participants without CL/P (Slifer et al., 2004; Hunt et al., 2006; Oosterkamp et al., 2007; Chuo et al., 2008; Mani et al., 2010; Versnel et al., 2010; Van Lierde et al., 2012).

Participants with cleft palate, with or without cleft lip (CP±L), have reported more negative attitudes toward communication (Havstam et al., 2011) and less satisfaction with overall speech (Hunt et al., 2006) than a control population without CP±L. Another study found no difference in satisfaction with speech between populations with and without UCLP (Van Lierde et al., 2012). Patients with BCLP have reported similar quantitative results on satisfaction with speech as a control population (Oosterkamp et al., 2007). However, in the same study, qualitative results revealed that participants with BCLP had significantly more concerns regarding articulation and nasality of speech than the control population.

In order to facilitate the interpretation of CHASQ scores in the CL/P population and to investigate content validity of CHASQ, the aims of this study were:

1. To compare CHASQ scores between patients with CL/P and a control population
2. To compare the CHASQ scores in this study, with the CHASQ scores of a British norm population with CL/P.

## Methods

### Cleft Lip and/or Cleft Palate Population

Data from 64 CL/P patients were collected on routine visits to a single CL/P center. Participants constituted a consecutive sample. They were given information about the study verbally as well as in written form. Participants did not receive any incentives for participation. Written consent was obtained from all participants. Participants younger than 15 years received written information about the study, which was adapted for their age group, and written consent was obtained from parents. Ethical approval was obtained from the Ethical Board in Lund, Sweden, reference nr: 2015/799. In total 151 questionnaires were handed out and 64 (42%) were returned; 71 questionnaires were given to patients to complete during their visit to the clinic, of these 49 (69%) were returned; 80 questionnaires were also given to the patients to take home to complete and return by post. Of these, 14 (18%) were retuned. Information on participant age, sex and type of CL/P was collected from medical journals. Age, sex, and distribution of types of CL/P are presented in Table 1.

**Table 1.** Sex, Age in Years, and Distribution of Types of Cleft Lip and/or Palate (CL/P) in the Study Populations.

Population	N	Sex	Mean age	SD	Min age	Max age	Cleft type
CL/P	64	25 girls (39%) 39 boys (61%)	13	3.6	7	19	CP 10 (15%) CL±A 23 (36%) UCLP 23 (36%) BCLP 8 (13%)
Control	56	30 girls (54%) 26 boys (46%)	13	3.3	9	20	–

Abbreviations: BCLP, bilateral cleft lip and palate; CL±A, cleft lip with or without cleft alveolus; CL/P, cleft lip and/or cleft palate; CP, cleft palate only; SD, standard deviation; UCLP, unilateral cleft lip and palate.

### Control Population

Fifty-six participants were recruited to the control population from a local sports club and a local school. Distribution of age and sex in the control population is presented in Table 1. All members in sports groups selected to match CL/P participant age were invited to take part in the study. All students in school classes selected to match CL/P participant age were invited to take part in the study. Participants were given information about the study verbally as well as in written form. Participants did not receive any incentives for participation. Written consent was obtained from all participants. Participants younger than 15 years received written information about the study, which was adapted for their age group, and written consent was obtained from parents. Two hundred twenty-four questionnaires were given to the control population to complete at home and return to the researcher at the school or sports club. Of these, 56 (25%) participants returned the questionnaire. There was no statistically significant difference in age (*P* = .8, *U* = 1,825.0) or sex (*P* = .1, *U* = 1,532.0) between the CL/P and the control population calculated with Mann-Whitney *U* test.

### Patient-Reported Outcome Measure—CHASQ

The PROM used in this study was the CHASQ, developed by the Cleft Psychology Clinical Excellence Network, 2015, see Online Appendix. It is a modified version of the Satisfaction with Appearance questionnaire (SWA), designed by the Cleft Psychology Special Interest Group, Craniofacial Society of Great Britain and Ireland, specifically for patients with facial disfigurement (Emerson et al., 2004). Satisfaction with Appearance and CHASQ have satisfactory internal validity, construct validity, and overall adequate psychometric properties, and both have been used in clinical research in Europe (Berger and Dalton, 2009; Feragen et al., 2009; Feragen and Borge, 2010; Mani et al., 2010; Mani et al., 2013; Feragen et al., 2015; Feragen and Stock, 2016; Crerand et al., 2017; Stiernman et al., 2019).

Cleft Hearing, Appearance and Speech Questionnaire consists of 9 items regarding features typically affected by a cleft (features 1) and 6 items regarding features not typically affected by a cleft (features 2), see Table 2. Therefore, CHASQ produces 2 scores, sum of total features 1 and sum of total features 2. The score for each item ranges from 0 to 10 points. Higher scores indicate higher satisfaction.

**Table 2.** Items in CHASQ, Divided into Features 1 or 2.^a^

CHASQ item	Features 1	Features 2
1. Face		
2. Whole appearance		
3. Side view/profile		
4. Good-looking		
5. Nose		
6. Lips		
7. Chin		
8. Teeth		
9. Cheeks		
10. Hair		
11. Ears		
12. Eyes		
13. Speech		
14. Hearing		
15. How noticeable to others		

Abbreviation: CHASQ, Cleft Hearing Appearance and Speech Questionnaire.

^a^ Adapted from CHASQ User Guide (Cleft Psychology Clinical Excellence Network, 2015).

Norm values of the CHASQ have been established by the Cleft Psychology Clinical Excellence Network in 2015 (User Guide - unpublished work) to aid in the interpretation of patient scores based on results from 867 patients with CL/P in the United Kingdom. The group consisted of 469 males and 398 females with CL/P, aged 10 (n = 457), 15 (n = 287), and 20 (n = 123) years of age. Types of CL/P represented were unilateral cleft lip with or without cleft alveolus (UCL±A; n = 169), UCLP (n = 280), bilateral cleft lip with or without cleft alveolus (n = 22), BCLP (n = 89), CP (n = 280), and submucous cleft palate (n = 27). Scores were defined as “less satisfied than expected” if the patient scored in the 15th percentile, and “very much less satisfied than expected” if the patients scored in the 5th percentile. The cutoffs were not related to average satisfaction with hearing, appearance, or speech in groups of people without CL/P. For the total features 1, the median score in the norm population was approximately 69 points, the 15th percentile cutoff approximately 50 points and the 5th percentile cutoff approximately 34 points. For the total features 2, the median was approximately 55 points, the 15th percentile cutoff approximately 44 points, and the 5th percentile cutoff approximately 36 points.

### Statistics

In this study, the scores of total features 1 and total features 2 were analysed separately. Participants in the Swedish control population did not answer item nr 15: *How visible do you think your cleft is to others?* Thus, item 15 was excluded from the total features 1 in the CL/P population in the comparison with the control population (comparison adjusted total features 1). Difference in CHASQ scores between the CL/P and control population was calculated with Mann-Whitney *U* test. Difference in CHASQ scores between cleft types was calculated with Kruskal-Wallis test. Spearman test was used to calculate correlations between CHASQ scores and age. For all statistical analyses, *P* < .05 (2 tailed) was considered to indicate a significant difference.

## Results

### Comparison Between CHASQ Scores in CL/P and Control Population

**Figure 1.**
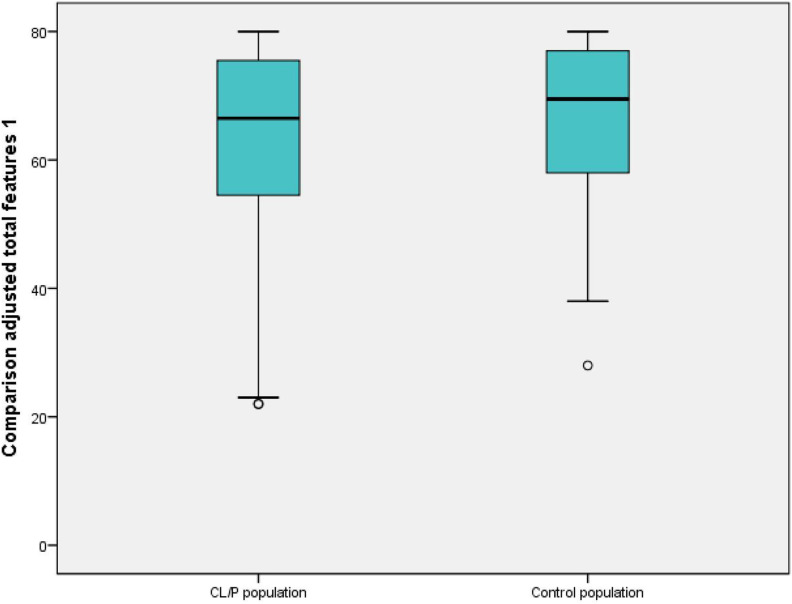
Boxplot showing results of comparison adjusted total features 1 in cleft lip and/or cleft palate (CL/P) population and control population. Median, range, and outliers (°) are presented. High scores indicate high satisfaction.

Results of the comparison adjusted total features 1 are presented in Figure 1 and Table 3. Higher scores indicated higher satisfaction; 80 points was the highest possible score. Scores for the CL/P population ranged from 22 to 80 points; median score was 67 points. Scores for the control population ranged from 28 to 80 points; median score was 67 points. There was no statistically significant difference between the CL/P and the control population calculated with Mann-Whitney *U* test (*P* = .3, *U* = 2000.5). Comparison adjusted total features 1 correlated moderately with age in the CL/P population (*P* = .001, r = −0.44) and in the control population (*P* = .001, r = −0.58) in Spearman test. Older age was associated with lower satisfaction. Comparison adjusted total features 1 showed no significant difference in the distribution between boys and girls in either the CL/P population (*P* = .8, *U* = 503.0) or the control population (*P* = .2, *U* = 469.5) in the Mann-Whitney *U* test.

**Table 3.** Median and Mean of Single Items of CHASQ, Comparison Adjusted Total Features 1 and Total of Features 2.^a^

CHASQ item	CL/P population score		Control population score		*U*	*P*
	Median	Mean	Median	Mean		
1. Face	8.0	8.0	9.0	8.4	1923.5	.4
2. Whole appearance	9.0	8.2	9.0	8.4	1840.5	.8
3. Side view/profile	9.0	7.9	9.0	8.3	1909.0	.5
4. Good-looking	8.0	7.7	8.5	8.0	1831.0	.8
5. Nose	9.0	7.5	9.0	8.3	2136.5	.06
6. Lips	9.0	7.6	9.5	8.9	2267.5	.009^b^
7. Chin	10.0	9.1	9.0	8.5	1308.5	.005^b^
8. Teeth	8.0	7.8	8.0	7.7	1789.0	1.0
9. Cheeks	10.0	9.0	9.0	8.7	1413.0	.03^b^
10. Hair	10.0	9.4	9.0	8.4	1228.0	.001^b^
11. Ears	10.0	9.1	10.0	8.8	1629.5	.3
12. Eyes	10.0	9.6	10.0	9.3	1611.5	.2
13. Speech	9.0	8.4	9.0	8.6	1880.5	.6
14. Hearing	10.0	8.9	9.5	8.9	1779.0	.9
15. How noticeable to others	8.0	7.4	-	-	-	-
Total features 1	75	71	-	-	-	-
Comparison adjusted total features 1	67	63	67	66	2000.5	.3
Total features 2	58	55	56	53	1367.0	.02^b^

Abbreviations: CHASQ, Cleft Hearing, Appearance and Speech Questionnaire; CL/P, cleft lip and/or cleft palate.

^a^ Differences between the CL/P and control population calculated with Mann-Whitney *U* test.

^b^ Significant difference at the 0.05 level.

**Figure 2.**
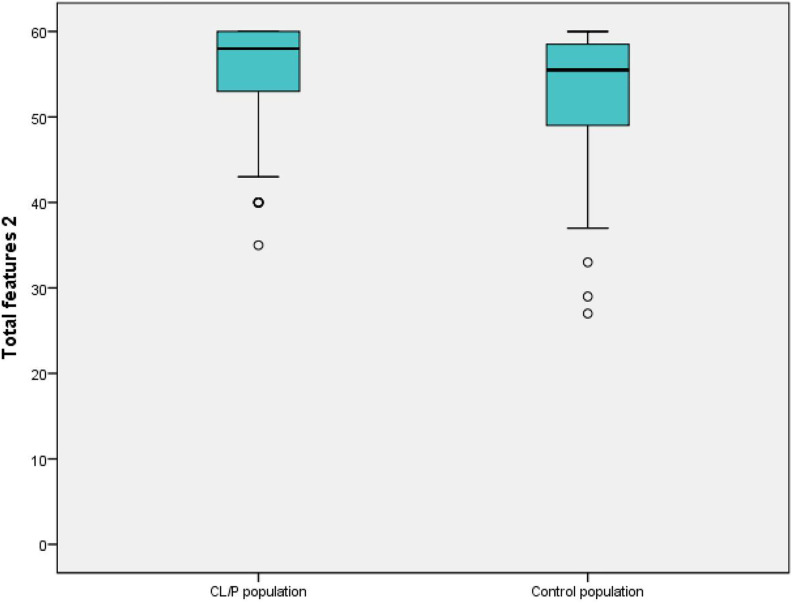
Boxplot showing results of total features 2 in cleft lip and/or cleft palate (CL/P) population and control population. Median, range, and outliers (°) are presented. High scores indicate high satisfaction.

Results of the total features 2 are presented in Figure 2 and Table 3. Higher scores indicated higher satisfaction; 60 points was the highest possible score. Scores from the CL/P population ranged from 35 to 60 points; median score was 58 points. Scores for the control population ranged from 27 to 60 points; median score was 56 points. There was a statistically significant difference in total features 2 between the CL/P and the control population calculated with Mann-Whitney *U* test (*P* = .02, *U* = 1,367.00). Higher age correlated weakly with lower score of total features 2 in the CL/P population (*P* = .07, r = −0.23) and moderately in the control population (*P* = .001, r = −0.51) in Spearman test. There was no significant difference in the distribution of total features 2 between boys and girls in either the CL/P population (*P* = .5, *U* = 434.5) or the control population (*P* = .1, *U* = 477.5) in the Mann-Whitney *U* test.

On a single item level, there was a significant difference between the CL/P and the control population on 4 items calculated with Mann-Whitney *U* test, see Table 3. The control population was more satisfied with the appearance of their lips. On the other hand, the CL/P population was more satisfied than the control population with the appearance of their chin, cheeks, and hair. There was no significant difference in the distribution of scores of the total features 1 (*P* = .8, χ^2^ = 1.18) or total features 2 (*P* = .4, χ^2^ = 3.08) between different cleft types with the Kruskal-Wallis test. Neither was there any difference in subscores of satisfaction with hearing (*P* = .3, χ^2^ = 3.45), appearance (*P* = .4, χ^2^ = 2.95), or speech (*P* = .4, χ^2^ = 3.04) between different cleft types.

### Comparison Between CHASQ Scores of the Swedish CL/P and Control Population and the British Norm Population With CL/P

In the Swedish CL/P population, the median score of total features 1 was 75, which corresponded to the median of 69 points in the British CHASQ norms presented by the Cleft Psychology Clinical Excellence Network in 2015. In total, 5 (8%) participants with CL/P scored less than 50 points, which corresponded to the 15th percentile. Four (6%) participants with CL/P scored less than 34 points, corresponding to the 5th percentile. In the CL/P population, the median score of total features 2 was 58, which corresponded to the median of 55 points in the British CHASQ norms. In total, 7 (13%) participants with CL/P scored less than 44, corresponding to the 15th percentile. Only 1 (2%) participant with CL/P scored less than 36 points, corresponding to the 5th percentile. In the control population, the median score of total features 2 was 56, 8 (14%) participants scored less than 44 points on the total features 2, and 3 (5%) scored less than 36 points.

## Discussion

### Cleft Hearing, Appearance and Speech Questionnaire Scores in CL/P and Control Population

There was no statistically significant difference between the CL/P and the control population on comparison adjusted total features 1, which represents satisfaction with appearance of features typically affected by a cleft. Although difference in the median score of the total features 2 was only 2 points, the CL/P population as a group reported significantly higher satisfaction with non-cleft-related features than the control population. Analysis on single item level revealed that the CL/P population was more satisfied than the control population with their chin, cheeks, and hair. Although a significant difference, the median CHASQ item score only differed by one point or less. The CL/P population was less satisfied with their lips. The difference in median lip score was 0.5 points. It is not clear if these differences are clinically significant.

These similar levels of satisfaction are in line with earlier publications on comparisons of SWA scores between CL/P population and control population (Berger and Dalton, 2009; Feragen et al., 2015; Crerand et al., 2017). An earlier comparison of Swedish children also showed similar levels of general HRQOL in the CL/P population as in a control population (Sundell et al., 2017). The results may reflect some confounding factors discussed in earlier studies, such as gratitude or unwillingness to disappoint cleft teams caring for them. Patients could also be influenced by the difference in appearance they have experienced through treatment, and consequently have reported satisfaction with the improvement in hearing, appearance, or speech, rather than with the end result (Crerand et al., 2017). Additional reasons for reporting high satisfaction may be acceptance of the current appearance and realistic expectations (Thomas et al., 1997; Oosterkamp et al., 2007) or lower levels of investment in appearance in the CL/P population compared to a control population (Stock et al., 2015; Crerand et al., 2017).

Even though subgroups of cleft type were small, there were no indications of different CHASQ scores between different types of cleft. This finding is also in line with some earlier research (Feragen et al., 2009; Berger and Dalton, 2011). Differences in PROs have, however, been found between cleft types in a study with larger sample sizes (Wong Riff et al., 2018).

The similarity in scores of total features 1 between the CL/P and control population indicates that participants in the control population were similarly affected by concerns with appearance, hearing, and speech. This was furthermore reflected in the percentage of the control population who fell into the categories of “less/very much less satisfied than expected” on total features 2. These findings are in accordance with earlier studies, which have reported widespread dissatisfaction with appearance in the adolescent and adult population in general (Versnel et al., 2010; Rumsey and Harcourt, 2012).

It is not clear from the results of this study whether low satisfaction with a facial feature affects other domains of psychological health in individuals with CL/P in the same way as those without CL/P. In patients with CL/P, for example, lower satisfaction with the lip may affect the perception of difference. This is a factor shown to influence psychological adjustment (Stock et al., 2015). In comparison, a person with low satisfaction with their chin, cheeks of hair may not perceive this feature as equally stigmatizing. Association has been found between dissatisfaction with appearance and elevated risk for low emotional adjustment in both 16-year-old CL/P and non-CL/P participants (Feragen et al., 2015). The risk of low adjustment, however, seemed to be specific to a domain rather than to HRQOL in general. For example, low satisfaction with appearance was associated with emotional adjustment, but did not spill over into other domains of adjustment such as cognitive, behavioral, or social functioning as exemplified in the study. The scope of this study was to explore the satisfaction of appearance, hearing, or speech within a CL/P and non-CL/P population. Association between level of satisfaction and other psychological domains was, however, outside the scope of this study.

### Cleft Hearing, Appearance and Speech Questionnaire Scores in Swedish and British CL/P Populations

The median Swedish CHASQ scores of both total features 1 and 2 were similar to the median scores in the British norm population (Cleft Psychology Clinical Excellence Network, 2015). The percentage of CL/P population in this study defined as “less/very much less satisfied than expected” was in line with the British norm population. This is an indication that the cutoffs of the questionnaire possibly work similarly in both the British and Swedish CL/P population. The percentage of the control population in this study, defined as “less/very much less satisfied than expected” regarding total features 2, was also in line with the British norm population. There was no control population included in the British normative data.

### Limitations of the Study

The small study populations and the loss of participants in the study pose a risk for inclusion bias and impede extrapolation of the results to other populations. There is a risk that children/young people who thought the questionnaire was psychologically challenging, due to dissatisfaction with their own appearance, did not fill out the questionnaire and therefore, could be underrepresented in both populations. No telephone follow-up to remind participants to return the questionnaires was carried out. This could, in part, explain the low participation rate. The low participation rate shows that handing out questionnaires, without a follow-up or reminder, is not an optimal method for collection of questionnaire data.

A qualitative analysis was not included. Even though an earlier study reported similar levels of satisfaction with hearing, appearance, and speech between CL/P and control population in a quantitative analysis, significant concerns were revealed in the CL/P population according to a qualitative analysis (Oosterkamp et al., 2007). Thus, an additional qualitative methodology could also have broadened the analysis in this study.

The raw data of the British norm population were not available to the authors. Detailed comparison between the total scores and single item scores between the British norm population and the Swedish populations could therefore not be performed.

## Conclusion

The results of this study indicate that children and young people with CL/P were as satisfied with their appearance, hearing, and speech as children and young people without CL/P. Cleft Hearing, Appearance and Speech Questionnaire scores were also similar to earlier established British normative data of a CL/P population.

## Supplemental Material

Supplemental Material, Appendix_CHASQ_(1) - Scores of the Cleft Hearing, Appearance and Speech Questionnaire (CHASQ) in Swedish Participants With Cleft lip and/or Cleft Palate and a Control Population Click here for additional data file. Supplemental Material, Appendix_CHASQ_(1) for Scores of the Cleft Hearing, Appearance and Speech Questionnaire (CHASQ) in Swedish Participants With Cleft lip and/or Cleft Palate and a Control Population by Mia Stiernman, Kristina Klintö, Martin Persson and Magnus Becker in The Cleft Palate-Craniofacial Journal
