# Supplementary material for: Scores of the Cleft Hearing, Appearance and Speech Questionnaire (CHASQ) in Swedish Participants With Cleft lip and/or Cleft Palate and a Control Population
Source: Cleft Palate Craniofac J. 2020 Sep 4;58(3):347–53. doi: 10.1177/1055665620952296 (PMC7874374; doi:10.1177/1055665620952296)
Supplement: Supplemental Material, Appendix_CHASQ_(1) - Scores of the Cleft Hearing, Appearance and Speech Questionnaire (CHASQ) in Swedish Participants With Cleft lip and/or Cleft Palate and a Control Population [file Appendix_CHASQ_(1).docx]

| Name | Date of Birth | Date completed |
| --- | --- | --- |
|  |  |  |

Some young people tell us that they are happy with their hearing, appearance and speech while others sometimes feel less happy.

How do you feel about your hearing, appearance and speech?

There are no right or wrong answers.

Please tick one box for each question.

**How happy are you with:**

**1. How your face looks:**

Very ☺ ☹ Very

happy unhappy

###### 10 0

**2. The whole of your appearance:**

Very ☺ ☹ Very

happy unhappy

###### 10 0

**3. Side view / profile:**

Very ☺ ☹ Very

happy unhappy

###### 10 0

**4. How good-looking do you think you are?**

Very ☺ ☹ Not at all

good-looking good- looking

###### 10 0

**How do you feel about these parts of your face?**

**5. Nose:**

Very ☺ ☹ Very

happy unhappy

###### 10 0

**6. Lips:**

Very ☺ ☹ Very

happy unhappy

###### 10 0

**7. Chin:**

Very ☺ ☹ Very

happy unhappy

###### 10 0

**8. Teeth:**

Very ☺ ☹ Very

happy unhappy

###### 10 0

**9. Cheeks:**

Very ☺ ☹ Very

happy unhappy

###### 10 0

**10. Hair:**

Very ☺ ☹ Very

happy unhappy

###### 10 0

**11. Ears:**

Very ☺ ☹ Very

happy unhappy

###### 10 0

**12. Eyes:**

Very ☺ ☹ Very

happy unhappy

###### 10 0

###### **13. How happy are you with your speech?**

Very ☺ ☹ Very

happy unhappy 10 0

###### **14. How happy are you with your hearing?**

Very ☺ ☹ Very

happy unhappy

###### 10 0

###### **15. Overall how noticeable do you feel your cleft is to other people?**

Not at all ☺ ☹ Very

noticeable noticeable

###### 10 0
